# Supplementary material for: Fluctuating reproductive rates in Hawaii's humpback whales, Megaptera novaeangliae, reflect recent climate anomalies in the North Pacific
Source: R Soc Open Sci. 2019 Mar 20;6(3):181463. doi: 10.1098/rsos.181463 (PMC6458358; doi:10.1098/rsos.181463)
Supplement: Supplementary Tables [file rsos181463supp1.docx]

Supplemental Data

Cartwright, R., A. Venema , V. Hernandez, C. Wyels, J. Cesere, D. Cesere. 2019. Fluctuating reproductive rates in Hawaii’s humpback whales, *Megaptera novaeangliae*, reflect recent climate anomalies in the North Pacific. Royal Society Open Science

Table S1: Waypoints in the Au’Au Channel

|  | Latitude  (Decimal degrees N) | Longitude  (Decimal degrees W) |
| --- | --- | --- |
| Waypoint 1 | 20.883333 | 156.768326 |
| Waypoint 2 | 20.883333 | 156.694168 |
| Waypoint 3 | 20.866667 | 156.766663 |
| Waypoint 4 | 20.866667 | 156.677505 |
| Waypoint 5 | 20.850000 | 156.759872 |
| Waypoint 6 | 20.850000 | 156.664673 |
| Waypoint 7 | 20.833333 | 156.756561 |
| Waypoint 8 | 20.833333 | 156.651337 |
| Waypoint 9 | 20.816668 | 156.739334 |
| Waypoint 10 | 20.816668 | 156.632828 |
| Waypoint 11 | 20.800000 | 156.700500 |
| Waypoint 12 | 20.800000 | 156.597504 |

Table S2: Group locations, with depths and distances from shore, derived from ArcGIS 10.3

| Pod ID | Year | Season | N of whales | Group Comp-  osition | Latitude | Longitude | Depth  (m) | Distance to shore(m) |
| --- | --- | --- | --- | --- | --- | --- | --- | --- |
| 31608A | 2008 | late | 3 | MCE | 20.82463300 | -156.70990000 | 67.97 | 5663.26 |
| 31608B | 2008 | late | 1 | S | 20.80140000 | -156.67203300 | 71.21 | 4488.08 |
| 31708A | 2008 | late | 2 | MC | 20.88298300 | -156.69566700 | 29.12 | 842.09 |
| 31708B | 2008 | late | 3 | MCE | 20.87336700 | -156.72850000 | 53.30 | 4354.30 |
| 31708C | 2008 | late | 4 | MCEE | 20.86346700 | -156.73026700 | 45.97 | 4882.00 |
| 31808A | 2008 | late | 4 | MCEE | 20.86635000 | -156.68925000 | 26.29 | 1265.28 |
| 31808B | 2008 | late | 2 | MC | 20.85198300 | -156.73033300 | 47.06 | 5541.37 |
| 31808C | 2008 | late | 1 | S | 20.83505000 | -156.70816700 | 65.38 | 4813.21 |
| 31808D | 2008 | late | 3 | MCE | 20.83515000 | -156.69893300 | 64.26 | 4036.94 |
| 32008A | 2008 | late | 3 | MCE | 20.84455000 | -156.72643300 | 58.72 | 5779.19 |
| 32008B | 2008 | late | 4 | MUL | 20.83995000 | -156.72918300 | 72.65 | 6315.09 |
| 32008C | 2008 | late | 4 | MUL | 20.83335000 | -156.73243300 | 74.90 | 7000.57 |
| 32008D | 2008 | late | 1 | S | 20.82291700 | -156.66668300 | 54.41 | 2376.17 |
| 32108A | 2008 | late | 3 | MCE | 20.87211700 | -156.74753300 | 73.74 | 6324.43 |
| 32108B | 2008 | late | 1 | S | 20.86938300 | -156.74876700 | 74.40 | 6508.77 |
| 32108C | 2008 | late | 3 | MCE | 20.88236700 | -156.74286700 | 63.14 | 5745.21 |
| 32108F | 2008 | late | 5 | MUL | 20.84635000 | -156.76120000 | 69.15 | 8606.99 |
| 32208A | 2008 | late | 1 | S | 20.82410000 | -156.68480000 | 64.04 | 3885.10 |
| 32208B | 2008 | late | 3 | MUL | 20.80496700 | -156.65851700 | 58.08 | 3326.45 |
| 32208C | 2008 | late | 2 | MC | 20.80923300 | -156.65408300 | 58.83 | 2747.48 |
| 32308A | 2008 | late | 3 | MCE | 20.88168300 | -156.73406700 | 43.75 | 4829.42 |
| 32308B | 2008 | late | 3 | MCE | 20.88070000 | -156.74178300 | 55.43 | 5633.84 |
| 32308C | 2008 | late | 3 | MCE | 20.86211700 | -156.72318300 | 73.81 | 4298.02 |
| 32408A | 2008 | late | 2 | MC | 20.83363300 | -156.68885000 | 52.31 | 3446.74 |
| 32408D | 2008 | late | 3 | MCE | 20.83710000 | -156.67216700 | 48.02 | 2002.45 |
| 32408E | 2008 | late | 4 | MUL | 20.85425000 | -156.74178300 | 70.97 | 6411.64 |
| 32508D | 2008 | late | 3 | MCE | 20.80233300 | -156.65201700 | 57.94 | 2829.84 |
| 32508E | 2008 | late | 1 | S | 20.81356700 | -156.69563300 | 55.12 | 5475.55 |
| 32508F | 2008 | late | 2 | D | 20.80610000 | -156.69975000 | 73.20 | 6273.40 |
| 32508G | 2008 | late | 1 | S | 20.80686700 | -156.67811700 | 71.41 | 4451.38 |
| 32508H | 2008 | late | 2 | MC | 20.82573300 | -156.68965000 | 61.77 | 4189.15 |
| 32508I | 2008 | late | 2 | MC | 20.82648300 | -156.70115000 | 73.33 | 4871.43 |
| 32608A | 2008 | late | 3 | MCE | 20.88023300 | -156.74658300 | 51.35 | 6134.52 |
| 32608B | 2008 | late | 2 | MC | 20.87143300 | -156.75573300 | 73.56 | 7178.41 |
| 32608C | 2008 | late | 3 | MCE | 20.84735000 | -156.72511700 | 54.40 | 5462.91 |
| 32608D | 2008 | late | 3 | MCE | 20.83675000 | -156.68140000 | 53.60 | 2731.71 |
| 32608E | 2008 | late | 1 | S | 20.83958300 | -156.67518300 | 44.80 | 2042.90 |
| 32608F | 2008 | late | 3 | MCE | 20.83188300 | -156.65601700 | 27.84 | 901.24 |
| 32708A | 2008 | late | 2 | D | 20.80133300 | -156.69551700 | 69.38 | 6212.28 |
| 32708B | 2008 | late | 3 | MCE | 20.79948300 | -156.70655000 | 80.74 | 7264.73 |
| 32708C | 2008 | late | 1 | S | 20.80311700 | -156.68983300 | 70.83 | 5627.89 |
| 32708D | 2008 | late | 2 | D | 20.80251700 | -156.68970000 | 70.39 | 5660.32 |
| 32708F | 2008 | late | 5 | MCEE | 20.80321700 | -156.68105000 | 72.89 | 4954.42 |
| 32708G | 2008 | late | 3 | MCE | 20.81560000 | -156.66060000 | 56.58 | 2516.54 |
| 31609A | 2009 | late | 2 | D | 20.83728300 | -156.72053300 | 71.75 | 5735.41 |
| 31609B | 2009 | late | 1 | S | 20.83833300 | -156.72773300 | 74.84 | 6284.92 |
| 31609C | 2009 | late | 2 | D | 20.83585000 | -156.74415000 | 66.46 | 7783.20 |
| 31609D | 2009 | late | 1 | S | 20.82793300 | -156.68348300 | 56.09 | 3552.73 |
| 31609E | 2009 | late | 1 | S | 20.82545000 | -156.67745000 | 50.87 | 3172.06 |
| 31609F | 2009 | late | 3 | MCE | 20.82398300 | -156.68003300 | 55.33 | 3484.36 |
| 31809A | 2009 | late | 1 | S | 20.82626700 | -156.67610000 | 64.79 | 3005.57 |
| 31809B | 2009 | late | 1 | S | 20.82456700 | -156.68268300 | 54.86 | 3672.16 |
| 31809C | 2009 | late | 1 | S | 20.81233300 | -156.67793300 | 72.31 | 4023.06 |
| 31809D | 2009 | late | 4 | MCEE | 20.81043300 | -156.67525000 | 66.62 | 3960.45 |
| 31909A | 2009 | late | 3 | MCE | 20.87806700 | -156.72431700 | 69.34 | 3838.60 |
| 31909C | 2009 | late | 3 | MCE | 20.84218300 | -156.71900000 | 62.47 | 5294.36 |
| 31909D | 2009 | late | 2 | MC | 20.83815000 | -156.69108300 | 52.95 | 3212.86 |
| 31909E | 2009 | late | 3 | MCE | 20.83743300 | -156.69306700 | 56.32 | 3415.23 |
| 32009A | 2009 | late | 3 | MCE | 20.87155000 | -156.70928300 | 56.27 | 2526.28 |
| 32009B | 2009 | late | 4 | MCEE | 20.82018300 | -156.68625000 | 62.86 | 4254.72 |
| 32109A | 2009 | late | 3 | MCE | 20.84908300 | -156.75536700 | 72.97 | 7928.75 |
| 32109C | 2009 | late | 1 | S | 20.83078300 | -156.72520000 | 78.11 | 6552.63 |
| 32109D | 2009 | late | 3 | MCE | 20.82103300 | -156.68805000 | 53.74 | 4354.05 |
| 32109E | 2009 | late | 8 | MUL | 20.82678300 | -156.68236700 | 53.34 | 3513.88 |
| 32309ABG | 2009 | late | 1 | S | 20.87280000 | -156.71638300 | 39.79 | 3155.12 |
| 32309BBG | 2009 | late | 4 | MCEE | 20.85291700 | -156.75146700 | 70.08 | 7375.87 |
| 32309CBG | 2009 | late | 4 | MCEE | 20.85350000 | -156.75375000 | 73.82 | 7563.37 |
| 32309DBG | 2009 | late | 3 | MCE | 20.85426700 | -156.75610000 | 73.86 | 7752.49 |
| 32309EBG | 2009 | late | 3 | MUL | 20.86686700 | -156.76835000 | 67.17 | 8560.66 |
| 32409A | 2009 | late | 1 | S | 20.81881700 | -156.71853300 | 75.11 | 6762.95 |
| 32409ABG | 2009 | late | 3 | MCE | 20.84186700 | -156.71035000 | 70.92 | 4579.41 |
| 32409B | 2009 | late | 2 | MC | 20.81456700 | -156.73503300 | 70.06 | 8403.25 |
| 32409BBG | 2009 | late | 3 | MCE | 20.84526700 | -156.73498300 | 53.10 | 6381.32 |
| 32409C | 2009 | late | 3 | MCE | 20.81495000 | -156.74345000 | 72.31 | 9105.99 |
| 32409CBG | 2009 | late | 1 | S | 20.83430000 | -156.74733300 | 60.71 | 8146.36 |
| 32409DBG | 2009 | late | 3 | MCE | 20.82718300 | -156.67926700 | 62.42 | 3216.02 |
| 32409EBG | 2009 | late | 3 | MCE | 20.80203300 | -156.64070000 | 52.00 | 1839.04 |
| 32509A | 2009 | late | 2 | D | 20.86283300 | -156.68543300 | 30.65 | 1121.09 |
| 32509ABG | 2009 | late | 3 | MCE | 20.85180000 | -156.73663300 | 69.60 | 6090.54 |
| 32509B | 2009 | late | 2 | MC | 20.86453300 | -156.70673300 | 54.20 | 2764.94 |
| 32509BBG | 2009 | late | 3 | MCE | 20.84908300 | -156.73605000 | 61.72 | 6211.02 |
| 32509CBG | 2009 | late | 2 | MC | 20.84900000 | -156.76641700 | 70.05 | 8969.93 |
| 32509D | 2009 | late | 2 | D | 20.87873300 | -156.75308300 | 48.27 | 6817.18 |
| 32509DBG | 2009 | late | 1 | S | 20.84576700 | -156.71681700 | 64.08 | 4883.25 |
| 32509E | 2009 | late | 2 | MC | 20.88461700 | -156.76833300 | 59.98 | 8400.04 |
| 32509EBG | 2009 | late | 3 | MCE | 20.84221700 | -156.68846700 | 49.69 | 2703.23 |
| 32509FBG | 2009 | late | 3 | MCE | 20.83416700 | -156.67225000 | 53.27 | 2197.84 |
| 32509GBG | 2009 | late | 2 | MC | 20.83526700 | -156.68333300 | 62.81 | 2986.21 |
| 32609A | 2009 | late | 1 | S | 20.81803300 | -156.65605000 | 52.00 | 2033.90 |
| 32609C | 2009 | late | 1 | S | 20.80318300 | -156.66105000 | 64.21 | 3642.17 |
| 32609D | 2009 | late | 3 | MCE | 20.81968300 | -156.67675000 | 65.63 | 3446.21 |
| 32609E | 2009 | late | 2 | D | 20.81806700 | -156.67323300 | 65.97 | 3243.87 |
| 32609F | 2009 | late | 3 | MCE | 20.81968300 | -156.67260000 | 64.08 | 3084.81 |
| 32609G | 2009 | late | 1 | S | 20.86783300 | -156.75956700 | 74.18 | 7643.71 |
| 32609H | 2009 | late | 3 | MCE | 20.85903300 | -156.70481700 | 43.92 | 3076.52 |
| 32709A | 2009 | late | 2 | MC | 20.81985000 | -156.74185000 | 79.64 | 8664.90 |
| 32709ABG | 2009 | late | 2 | D | 20.85201700 | -156.75268300 | 73.53 | 7533.26 |
| 32709B | 2009 | late | 3 | MCE | 20.83370000 | -156.66023300 | 36.97 | 1175.42 |
| 32709BBG | 2009 | late | 1 | S | 20.84538300 | -156.68975000 | 46.04 | 2584.70 |
| 32709CBG | 2009 | late | 4 | MUL | 20.86678300 | -156.76681700 | 68.59 | 8406.10 |
| 32709D | 2009 | late | 2 | D | 20.83681700 | -156.66876700 | 43.43 | 1746.12 |
| 32709DBG | 2009 | late | 1 | S | 20.85435000 | -156.70325000 | 45.05 | 3181.53 |
| 32709E | 2009 | late | 3 | MCE | 20.84386700 | -156.70120000 | 71.16 | 3695.16 |
| 32809A | 2009 | late | 4 | MCEE | 20.82791700 | -156.73630000 | 80.15 | 7674.72 |
| 32809ABG | 2009 | late | 1 | S | 20.81230000 | -156.65341700 | 56.46 | 2513.75 |
| 32809B | 2009 | late | 1 | S | 20.85845000 | -156.70448300 | 47.19 | 3074.48 |
| 32809BBG | 2009 | late | 1 | S | 20.80706700 | -156.65176700 | 57.88 | 2586.47 |
| 32809C | 2009 | late | 3 | MCE | 20.82896700 | -156.76015000 | 53.38 | 9554.39 |
| 32809CBG | 2009 | late | 2 | MC | 20.81681700 | -156.64200000 | 36.89 | 1325.05 |
| 32809DBG | 2009 | late | 2 | MC | 20.85281700 | -156.71966700 | 69.22 | 4634.25 |
| 32809EBG | 2009 | late | 1 | S | 20.86850000 | -156.71918300 | 68.24 | 3601.31 |
| 32809FBG | 2009 | late | 1 | S | 20.85183300 | -156.71918300 | 71.94 | 4675.16 |
| 32909A | 2009 | late | 3 | MCE | 20.86871700 | -156.68048300 | 6.85 | 332.50 |
| 32909ABG | 2009 | late | 3 | MCE | 20.86385000 | -156.74121700 | 67.96 | 5921.64 |
| 32909B | 2009 | late | 2 | D | 20.84833300 | -156.69191700 | 43.08 | 2609.87 |
| 32909BBG | 2009 | late | 3 | MUL | 20.86316700 | -156.74600000 | 73.64 | 6416.39 |
| 32909C | 2009 | late | 3 | MCE | 20.84883300 | -156.68933300 | 44.56 | 2345.73 |
| 32909CBG | 2009 | late | 1 | S | 20.86371700 | -156.74726700 | 74.78 | 6522.20 |
| 32909D | 2009 | late | 3 | MCE | 20.83600000 | -156.71360000 | 56.33 | 5243.43 |
| 32909DBG | 2009 | late | 1 | S | 20.83536700 | -156.70460000 | 59.99 | 4486.94 |
| 32909E | 2009 | late | 3 | MCE | 20.85315000 | -156.67685000 | 35.82 | 995.07 |
| 32010A | 2010 | late | 3 | MCE | 20.83471700 | -156.75170000 | 59.93 | 8469.36 |
| 32010B | 2010 | late | 4 | MUL | 20.82885000 | -156.70088300 | 68.90 | 4662.20 |
| 32010C | 2010 | late | 3 | MCE | 20.80555000 | -156.66498300 | 67.27 | 3696.74 |
| 32010D | 2010 | late | 2 | MC | 20.82001700 | -156.64996700 | 46.00 | 1595.20 |
| 32110a | 2010 | late | 3 | MCE | 20.83280000 | -156.66076700 | 39.67 | 1277.08 |
| 32110B | 2010 | late | 1 | S | 20.83408300 | -156.67666700 | 56.94 | 2569.40 |
| 32110C | 2010 | late | 1 | S | 20.84275000 | -156.72036700 | 59.26 | 5375.83 |
| 32110D | 2010 | late | 1 | S | 20.84275000 | -156.73190000 | 60.41 | 6324.81 |
| 32210A | 2010 | late | 3 | MCE | 20.85640000 | -156.70691700 | 49.97 | 3402.77 |
| 32210B | 2010 | late | 3 | MCE | 20.85425000 | -156.72696700 | 50.35 | 5110.52 |
| 32210C | 2010 | late | 3 | MCE | 20.86180000 | -156.73051700 | 46.76 | 4985.33 |
| 32210D | 2010 | late | 1 | S | 20.86301700 | -156.73303300 | 56.16 | 5164.82 |
| 32210E | 2010 | late | 3 | MCE | 20.86961700 | -156.74978300 | 75.01 | 6606.71 |
| 32210F | 2010 | late | 2 | MC | 20.86666700 | -156.76666700 | 70.19 | 8393.38 |
| 32210G | 2010 | late | 2 | MC | 20.87171700 | -156.75783300 | 73.54 | 7389.26 |
| 32310A | 2010 | late | 2 | D | 20.86731700 | -156.76493300 | 69.12 | 8202.21 |
| 32310B | 2010 | late | 1 | S | 20.86965000 | -156.75656700 | 74.14 | 7298.24 |
| 32310C | 2010 | late | 3 | MCE | 20.87261700 | -156.75183300 | 74.54 | 6757.01 |
| 32310D | 2010 | late | 1 | S | 20.87216700 | -156.75478300 | 73.27 | 7068.03 |
| 32310E | 2010 | late | 4 | MCEE | 20.87086700 | -156.74898300 | 74.53 | 6497.78 |
| 32310F | 2010 | late | 3 | MCE | 20.87476700 | -156.72906700 | 34.97 | 4381.25 |
| 32310G | 2010 | late | 3 | MCE | 20.87645000 | -156.71896700 | 53.05 | 3313.92 |
| 32410A | 2010 | late | 3 | MCE | 20.80538300 | -156.64975000 | 54.32 | 2464.19 |
| 32410C | 2010 | late | 3 | MCE | 20.81131700 | -156.67640000 | 72.15 | 3978.40 |
| 32410D | 2010 | late | 1 | S | 20.80891700 | -156.69955000 | 74.27 | 6097.31 |
| 32410E | 2010 | late | 2 | D | 20.81028300 | -156.70175000 | 58.15 | 6204.41 |
| 32410F | 2010 | late | 3 | MCE | 20.81090000 | -156.70591700 | 94.70 | 6524.45 |
| 11813a | 2013 | early | 1 | S | 20.88161300 | -156.76774600 | 53.45 | 8333.78 |
| 11813b | 2013 | early | 2 | D | 20.88041300 | -156.77084400 | 47.81 | 8657.70 |
| 11813c | 2013 | early | 2 | D | 20.88021700 | -156.76205400 | 44.64 | 7743.77 |
| 11813d | 2013 | early | 3 | MCE | 20.87644000 | -156.75816300 | 47.92 | 7361.71 |
| 11813e | 2013 | early | 3 | MCE | 20.88285800 | -156.76210000 | 61.60 | 7746.91 |
| 11813f | 2013 | early | 3 | MCE | 20.88208800 | -156.77034000 | 54.89 | 8603.64 |
| 11813i | 2013 | early | 2 | D | 20.88428300 | -156.75824000 | 57.80 | 7349.21 |
| 11813j | 2013 | early | 2 | D | 20.87642900 | -156.74806200 | 69.79 | 6314.94 |
| 11813k | 2013 | early | 3 | D | 20.87207600 | -156.74157700 | 65.51 | 5715.72 |
| 11813l | 2013 | early | 2 | D | 20.87033700 | -156.72563200 | 72.03 | 4154.52 |
| 11913a | 2013 | early | 2 | MC | 20.83673500 | -156.69006300 | 58.37 | 3255.93 |
| 12113a | 2013 | early | 3 | MCE | 20.84237700 | -156.67068500 | 51.50 | 1488.83 |
| 12113b | 2013 | early | 3 | MCE | 20.83461000 | -156.68742400 | 55.93 | 3272.73 |
| 12113c | 2013 | early | 2 | MC | 20.83758700 | -156.68876600 | 55.34 | 3096.36 |
| 12113d | 2013 | early | 3 | MCE | 20.82945300 | -156.69488500 | 69.25 | 4196.17 |
| 12113e | 2013 | early | 3 | MCE | 20.82697300 | -156.71377600 | 74.08 | 5807.68 |
| 12113f | 2013 | early | 3 | MCE | 20.82684700 | -156.72049000 | 51.52 | 6384.11 |
| 122313d | 2013 | early | 2 | MC | 20.82092700 | -156.64198300 | 33.25 | 1028.03 |
| 12313a | 2013 | early | 2 | MC | 20.83378400 | -156.70153800 | 60.22 | 4341.63 |
| 12313b | 2013 | early | 3 | MCE | 20.82549300 | -156.71348600 | 73.61 | 5885.34 |
| 12313c | 2013 | early | 2 | MC | 20.81802200 | -156.64302100 | 37.36 | 1345.87 |
| 12313e | 2013 | early | 3 | MCE | 20.83020200 | -156.67788700 | 66.70 | 2921.20 |
| 12313f | 2013 | early | 2 | D | 20.84478400 | -156.71420300 | 52.58 | 4720.10 |
| 12513e | 2013 | early | 1 | S | 20.85064300 | -156.74356100 | 70.03 | 6770.11 |
| 12613a | 2013 | early | 2 | MC | 20.85029600 | -156.72622700 | 57.97 | 5326.07 |
| 12613b | 2013 | early | 3 | MUL | 20.86917300 | -156.72169500 | 69.94 | 3812.75 |
| 12613c | 2013 | early | 3 | MCE | 20.86713800 | -156.71962000 | 69.48 | 3706.84 |
| 12613d | 2013 | early | 3 | MCE | 20.85231800 | -156.74375900 | 71.03 | 6695.10 |
| 12613f | 2013 | early | 1 | S | 20.84461400 | -156.74276700 | 66.98 | 7067.44 |
| 12613g | 2013 | early | 1 | S | 20.84470700 | -156.76399200 | 71.64 | 8948.66 |
| 12613h | 2013 | early | 3 | MCE | 20.86098100 | -156.70846600 | 52.58 | 3172.04 |
| 13013a | 2013 | early | 3 | MCE | 20.83886700 | -156.74606300 | 63.38 | 7724.15 |
| 13013b | 2013 | early | 3 | MCE | 20.86380200 | -156.74337800 | 71.35 | 6135.50 |
| 13013c | 2013 | early | 3 | MCE | 20.86707300 | -156.76864600 | 67.17 | 8586.47 |
| 13013d | 2013 | early | 4 | MCEE | 20.86324300 | -156.75793500 | 76.11 | 7599.87 |
| 13013e | 2013 | early | 2 | D | 20.85607700 | -156.73696900 | 69.15 | 5875.21 |
| 13013f | 2013 | early | 2 | D | 20.86364700 | -156.75564600 | 75.76 | 7358.52 |
| 2213a | 2013 | early | 2 | D | 20.85208700 | -156.73481800 | 68.48 | 5915.58 |
| 2213b | 2013 | early | 5 | MUL | 20.84836000 | -156.73492400 | 60.37 | 6164.31 |
| 2213c | 2013 | early | 5 | MCEE | 20.86257000 | -156.72976700 | 42.65 | 4877.59 |
| 2213d | 2013 | early | 2 | MC | 20.85750600 | -156.72480800 | 73.63 | 4717.09 |
| 2213e | 2013 | early | 5 | MCEE | 20.85990000 | -156.72422800 | 69.58 | 4519.44 |
| 2213f | 2013 | early | 2 | MC | 20.84890000 | -156.71020500 | 72.42 | 4118.35 |
| 2213h | 2013 | early | 2 | D | 20.84713700 | -156.69895900 | 64.92 | 3290.58 |
| 2513a | 2013 | early | 2 | MC | 20.79714000 | -156.60531600 | 34.33 | 1106.02 |
| 219113d | 2013 | mid | 2 | D | 20.85019900 | -156.71090700 | 71.78 | 4100.60 |
| 21913a | 2013 | mid | 3 | MCE | 20.82590700 | -156.65968300 | 48.05 | 1604.07 |
| 21913b | 2013 | mid | 1 | S | 20.84044100 | -156.67646800 | 45.61 | 2077.20 |
| 21913c | 2013 | mid | 2 | D | 20.84151300 | -156.69056700 | 52.36 | 2917.55 |
| 21913e | 2013 | mid | 3 | MCE | 20.85765300 | -156.72647100 | 64.39 | 4850.91 |
| 21913f | 2013 | mid | 3 | MCE | 20.85729200 | -156.73820500 | 70.30 | 5924.78 |
| 21913g | 2013 | mid | 1 | S | 20.86157800 | -156.74267600 | 72.00 | 6152.33 |
| 21913h | 2013 | mid | 1 | S | 20.84898600 | -156.74871800 | 72.82 | 7326.05 |
| 21913i | 2013 | mid | 1 | S | 20.84564000 | -156.74101300 | 62.24 | 6852.94 |
| 21913j | 2013 | mid | 1 | S | 20.84193800 | -156.73684700 | 64.05 | 6767.35 |
| 21913k | 2013 | mid | 2 | MC | 20.83742700 | -156.71551500 | 66.49 | 5305.21 |
| 21913l | 2013 | mid | 3 | MCE | 20.84009700 | -156.70925900 | 65.44 | 4612.83 |
| 22013a | 2013 | mid | 3 | MCE | 20.83301900 | -156.70185900 | 56.35 | 4421.75 |
| 22013b | 2013 | mid | 1 | S | 20.82991200 | -156.68594400 | 55.74 | 3592.09 |
| 22013c | 2013 | mid | 2 | D | 20.81616800 | -156.64169300 | 36.73 | 1321.25 |
| 22013d | 2013 | mid | 2 | MC | 20.85063600 | -156.66937300 | 25.42 | 760.49 |
| 22013e | 2013 | mid | 2 | D | 20.85878400 | -156.71302800 | 68.20 | 3678.33 |
| 22013f | 2013 | mid | 3 | MCE | 20.86026600 | -156.71838400 | 71.07 | 3996.30 |
| 22313a | 2013 | mid | 2 | D | 20.86375600 | -156.72283900 | 72.52 | 4176.95 |
| 22313b | 2013 | mid | 1 | S | 20.87231600 | -156.73117100 | 36.04 | 4651.19 |
| 22313c | 2013 | mid | 2 | D | 20.87917700 | -156.74221800 | 35.68 | 5685.61 |
| 22313d | 2013 | mid | 1 | S | 20.87608100 | -156.74261500 | 63.74 | 5755.25 |
| 22313e | 2013 | mid | 4 | MCEE | 20.80862200 | -156.64329500 | 52.00 | 1703.64 |
| 22313f | 2013 | mid | 1 | S | 20.81711800 | -156.68170200 | 68.06 | 4034.32 |
| 22313ft2 | 2013 | mid | 3 | MCE | 20.83420400 | -156.66464200 | 42.86 | 1525.12 |
| 22313g | 2013 | mid | 2 | D | 20.82209400 | -156.68577600 | 48.01 | 4092.16 |
| 22313h | 2013 | mid | 3 | MCE | 20.81592900 | -156.68504300 | 60.56 | 4400.88 |
| 22413c | 2013 | mid | 1 | S | 20.81849700 | -156.68731700 | 72.57 | 4455.20 |
| 22413d | 2013 | mid | 2 | MC | 20.81793400 | -156.69433600 | 63.96 | 5089.23 |
| 22413d | 2013 | mid | 2 | MC | 20.81793400 | -156.69433600 | 63.96 | 5089.23 |
| 22413e | 2013 | mid | 3 | MCE | 20.82042100 | -156.69145200 | 51.82 | 4688.05 |
| 22413f | 2013 | mid | 2 | MC | 20.82412100 | -156.71186800 | 73.49 | 5854.10 |
| 22413g | 2013 | mid | 2 | MC | 20.82689300 | -156.71194500 | 72.59 | 5663.20 |
| 22513c | 2013 | mid | 3 | MUL | 20.83029200 | -156.72818000 | 78.29 | 6833.20 |
| 22513e | 2013 | mid | 2 | D | 20.82597700 | -156.71350100 | 72.10 | 5853.04 |
| 22513f | 2013 | mid | 3 | MCE | 20.82723200 | -156.71720900 | 76.03 | 6078.50 |
| 22813a | 2013 | mid | 3 | MCE | 20.83112900 | -156.75247200 | 57.93 | 8781.69 |
| 3113b | 2013 | mid | 3 | MCE | 20.82549500 | -156.69461100 | 69.86 | 4528.48 |
| 3213a | 2013 | mid | 2 | MC | 20.88165900 | -156.75149500 | 40.04 | 6642.83 |
| 3213b | 2013 | mid | 2 | D | 20.88106300 | -156.76422100 | 47.94 | 7967.51 |
| 3213d | 2013 | mid | 1 | S | 20.88164700 | -156.76475500 | 48.88 | 8022.55 |
| 3213g | 2013 | mid | 2 | D | 20.82228900 | -156.66828900 | 57.66 | 2551.99 |
| 3213h | 2013 | mid | 7 | MUL | 20.81371700 | -156.66735800 | 64.40 | 3129.12 |
| 3213i | 2013 | mid | 2 | D | 20.81316000 | -156.66507000 | 62.42 | 3015.67 |
| 3213j | 2013 | mid | 2 | MC | 20.81315200 | -156.66413900 | 63.92 | 2955.09 |
| 3213k | 2013 | mid | 3 | MCE | 20.80976300 | -156.65448000 | 59.19 | 2771.56 |
| 3213m | 2013 | mid | 3 | MCE | 20.83858500 | -156.66368100 | 33.38 | 1182.52 |
| 3191300 | 2013 | late | 1 | S | 20.84114600 | -156.73545800 | 64.27 | 6717.29 |
| 3201300 | 2013 | late | 3 | MCE | 20.85757100 | -156.69934100 | 51.82 | 2647.68 |
| 3221300 | 2013 | late | 1 | S | 20.86940600 | -156.68643200 | 21.52 | 863.93 |
| 31813a2 | 2013 | late | 3 | MCE | 20.82445300 | -156.67741400 | 56.78 | 3231.50 |
| 31813b2 | 2013 | late | 2 | MC | 20.84857000 | -156.68225100 | 42.05 | 1751.74 |
| 31813c2 | 2013 | late | 2 | MC | 20.86918100 | -156.70661900 | 41.55 | 2425.28 |
| 31813d2 | 2013 | late | 2 | MC | 20.84423300 | -156.67703200 | 44.42 | 1802.90 |
| 31913a2 | 2013 | late | 3 | MCE | 20.87328700 | -156.71756000 | 42.75 | 3255.16 |
| 31913b2 | 2013 | late | 3 | MCE | 20.86497500 | -156.74223300 | 71.27 | 5981.02 |
| 31913c2 | 2013 | late | 4 | MUL | 20.85617100 | -156.75419600 | 74.51 | 7488.07 |
| 31913d2 | 2013 | late | 5 | MUL | 20.84521900 | -156.74182100 | 64.92 | 6948.41 |
| 32013a2 | 2013 | late | 2 | MC | 20.86474600 | -156.68707300 | 36.94 | 1144.43 |
| 32013b2 | 2013 | late | 3 | MCE | 20.86528200 | -156.69523600 | 44.52 | 1882.51 |
| 32013c2 | 2013 | late | 3 | MCE | 20.86428800 | -156.69612100 | 48.22 | 2009.76 |
| 32013d2 | 2013 | late | 4 | MCEE | 20.86245700 | -156.70108000 | 56.04 | 2563.92 |
| 32013f2 | 2013 | late | 3 | MCE | 20.85507000 | -156.70771800 | 61.53 | 3543.84 |
| 32013g2 | 2013 | late | 3 | MCE | 20.83827200 | -156.74041700 | 59.80 | 7314.18 |
| 32013h2 | 2013 | late | 1 | S | 20.83588200 | -156.75067100 | 62.76 | 8305.56 |
| 32013I2 | 2013 | late | 2 | MC | 20.83461400 | -156.75988800 | 75.13 | 9161.57 |
| 32213a2 | 2013 | late | 3 | MCE | 20.85189600 | -156.66970800 | 21.87 | 679.78 |
| 32213b2 | 2013 | late | 3 | MCE | 20.86114500 | -156.71611000 | 69.51 | 3749.13 |
| 32213c2 | 2013 | late | 2 | MC | 20.84759900 | -156.67831400 | 41.58 | 1551.22 |
| 32213d2 | 2013 | late | 1 | S | 20.85744500 | -156.68551600 | 31.17 | 1485.26 |
| 11814a | 2014 | early | 2 | D | 20.83712200 | -156.74623100 | 59.99 | 7858.41 |
| 12314a | 2014 | early | 2 | MC | 20.80743000 | -156.63276700 | 29.29 | 822.72 |
| 12314b | 2014 | early | 2 | D | 20.81707000 | -156.68327300 | 65.72 | 4178.19 |
| 12414b | 2014 | early | 3 | MCE | 20.81969600 | -156.71849100 | 75.50 | 6696.15 |
| 12514a | 2014 | early | 1 | S | 20.85767700 | -156.69386300 | 44.73 | 2167.23 |
| 12514a | 2014 | early | 1 | S | 20.85767700 | -156.69386300 | 44.73 | 2167.23 |
| 12514b | 2014 | early | 2 | D | 20.83653100 | -156.74833700 | 60.81 | 8070.20 |
| 12514c | 2014 | early | 3 | MCE | 20.85894600 | -156.70031700 | 53.16 | 2662.56 |
| 22014a | 2014 | mid | 3 | MCE | 20.83477800 | -156.69426000 | 63.46 | 3711.05 |
| 22014b | 2014 | mid | 3 | MCE | 20.83213600 | -156.71266200 | 69.59 | 5381.39 |
| 22014d | 2014 | mid | 3 | MCE | 20.83304800 | -156.71138000 | 64.81 | 5214.42 |
| 22014e | 2014 | mid | 1 | S | 20.83229300 | -156.71569800 | 70.13 | 5638.75 |
| 22014f | 2014 | mid | 3 | MCE | 20.82794400 | -156.73797600 | 79.76 | 7816.13 |
| 22314a | 2014 | mid | 3 | MCE | 20.80218700 | -156.69978300 | 67.99 | 6513.84 |
| 22314b | 2014 | mid | 2 | D | 20.80473700 | -156.69512900 | 73.49 | 5955.08 |
| 22314c | 2014 | mid | 3 | MCE | 20.81037700 | -156.68429600 | 69.01 | 4675.96 |
| 22714a | 2014 | mid | 2 | D | 20.88051200 | -156.70410200 | 26.56 | 1718.47 |
| 22714b | 2014 | mid | 3 | MCE | 20.87190100 | -156.72181700 | 70.09 | 3723.70 |
| 22714c | 2014 | mid | 3 | MCE | 20.87040500 | -156.72160300 | 69.95 | 3755.39 |
| 22714d | 2014 | mid | 3 | MCE | 20.86236600 | -156.73793000 | 66.07 | 5661.67 |
| 22714e | 2014 | mid | 1 | S | 20.86282500 | -156.73486300 | 61.97 | 5347.85 |
| 22714f | 2014 | mid | 8 | MUL | 20.85391000 | -156.74765000 | 73.94 | 6970.78 |
| 32314a | 2014 | late | 2 | MC | 20.83635900 | -156.68493700 | 54.72 | 2967.84 |
| 32314b | 2014 | late | 1 | S | 20.82025900 | -156.71936000 | 76.07 | 6725.46 |
| 32314d | 2014 | late | 3 | MCE | 20.80745500 | -156.69032300 | 72.25 | 5373.02 |
| 32314e | 2014 | late | 3 | MCE | 20.80991400 | -156.68843100 | 70.96 | 5055.22 |
| 32314f | 2014 | late | 1 | S | 20.83568400 | -156.70684800 | 60.71 | 4659.95 |
| 32414a | 2014 | late | 3 | MCE | 20.88019900 | -156.70233200 | 28.17 | 1539.03 |
| 32614a | 2014 | late | 2 | MC | 20.86842700 | -156.71844500 | 67.71 | 3534.90 |
| 32714b | 2014 | late | 1 | S | 20.88090700 | -156.75398300 | 57.88 | 6902.57 |
| 32714c | 2014 | late | 3 | MCE | 20.88231100 | -156.75859100 | 45.53 | 7381.23 |
| 32714e | 2014 | late | 2 | MC | 20.88056600 | -156.75018300 | 50.48 | 6507.99 |
| 32714f | 2014 | late | 3 | MCE | 20.86491200 | -156.72216800 | 71.25 | 4054.89 |
| 32714g | 2014 | late | 2 | MC | 20.86458800 | -156.69886800 | 51.88 | 2245.37 |
| 31916a | 2016 | late | 3 | MCE | 20.83370000 | -156.69784500 | 61.99 | 4060.29 |
| 32016a | 2016 | late | 1 | S | 20.87470100 | -156.75885000 | 62.25 | 7451.17 |
| 32116a | 2016 | late | 2 | MC | 20.83714900 | -156.75079300 | 67.77 | 8230.34 |
| 32316b | 2016 | late | 2 | MC | 20.81293900 | -156.70359800 | 53.01 | 6199.39 |
| 11517a | 2017 | early | 3 | MCE | 20.85021600 | -156.75125100 | 74.04 | 7491.05 |
| 11517c | 2017 | early | 2 | D | 20.85560400 | -156.73492400 | 65.86 | 5716.79 |
| 11517d | 2017 | early | 1 | S | 20.85737200 | -156.73323100 | 57.89 | 5466.00 |
| 11617a | 2017 | early | 2 | D | 20.79937700 | -156.61592100 | 30.46 | 900.63 |
| 11617c | 2017 | early | 2 | D |  |  | 64.42 | 3292.01 |
| 11717a | 2017 | early | 3 | MUL | 20.82147800 | -156.69877600 | 74.13 | 5142.81 |
| 11717b | 2017 | early | 3 | MUL | 20.81745300 | -156.73741100 | 73.44 | 8427.17 |
| 11717c | 2017 | early | 2 | D | 20.82668500 | -156.71817000 | 76.60 | 6195.15 |
| 11917a | 2017 | early | 3 | MUL | 20.87555900 | -156.73643500 | 50.62 | 5124.39 |
| 11917b | 2017 | early | 1 | S | 20.87103300 | -156.73362700 | 46.45 | 4933.12 |
| 11917c | 2017 | early | 1 | S | 20.86903200 | -156.73536700 | 54.33 | 5165.65 |
| 11917d | 2017 | early | 3 | MUL | 20.87277400 | -156.73309300 | 43.10 | 4835.24 |
| 11917e | 2017 | early | 1 | S | 20.84516100 | -156.71850600 | 57.69 | 5067.26 |
| 11917F | 2017 | early | 2 | D | 20.83862700 | -156.75228900 | 70.51 | 8259.11 |
| 21517a | 2017 | mid | 2 | D | 20.86017000 | -156.72222900 | 72.71 | 4329.02 |
| 21517b | 2017 | mid | 2 | D | 20.84934400 | -156.72999600 | 54.88 | 5693.74 |
| 21517c | 2017 | mid | 2 | D | 20.85918200 | -156.72294600 | 68.34 | 4452.15 |
| 21517d | 2017 | mid | 3 | MCE | 20.86023700 | -156.71415700 | 65.25 | 3655.98 |
| 21517e | 2017 | mid | 6 | MUL | 20.85453800 | -156.70855700 | 66.62 | 3648.52 |
| 21517f | 2017 | mid | 1 | S | 20.85355600 | -156.70738200 | 65.28 | 3594.26 |
| 21517g | 2017 | mid | 3 | MCE | 20.84590300 | -156.67997700 | 42.73 | 1805.22 |
| 21717a | 2017 | mid | 4 | MCEE | 20.87791600 | -156.76547200 | 50.17 | 8109.30 |
| 21717B | 2017 | mid | 1 | S | 20.87924200 | -156.76393100 | 50.89 | 7942.34 |
| 21717c | 2017 | mid | 3 | MUL | 20.87874800 | -156.75842300 | 60.54 | 7372.05 |
| 21717d | 2017 | mid | 3 | MCE | 20.86203600 | -156.69352700 | 43.36 | 1877.75 |
| 22117A | 2017 | mid | 3 | MCE | 20.83260200 | -156.72331200 | 77.46 | 6274.21 |
| 22117B | 2017 | mid | 1 | S | 20.82452600 | -156.72161900 | 71.51 | 6626.31 |
| 22117C | 2017 | mid | 1 | S | 20.81881700 | -156.73744200 | 78.28 | 8348.61 |
| 22217A | 2017 | mid | 2 | D | 20.81165500 | -156.69455000 | 64.58 | 5495.00 |
| 22217b | 2017 | mid | 2 | MC | 20.82769600 | -156.66317700 | 49.46 | 1778.36 |
| 22217c | 2017 | mid | 2 | MC | 20.82732000 | -156.66241500 | 49.14 | 1732.57 |
| 22217D | 2017 | mid | 2 | MC | 20.82869700 | -156.65667700 | 36.85 | 1164.61 |
| 22217E | 2017 | mid | 3 | MCE | 20.82864600 | -156.65414400 | 30.45 | 983.92 |
| 22217f | 2017 | mid | 3 | MCE | 20.83137100 | -156.65551800 | 27.60 | 886.26 |
| 32017A | 2017 | late | 3 | MCE | 20.87978000 | -156.76780700 | 49.87 | 8343.46 |
| 32017B | 2017 | late | 1 | S | 20.87709600 | -156.75540200 | 50.66 | 7069.56 |
| 32017c | 2017 | late | 3 | MCE | 20.87143500 | -156.72602800 | 71.64 | 4158.17 |
| 32017d | 2017 | late | 2 | MC | 20.86443500 | -156.71775800 | 70.88 | 3682.14 |
| 32017E | 2017 | late | 2 | MC | 20.85905500 | -156.70050000 | 53.16 | 2674.10 |
| 32117e Ak | 2017 | late | 2 | MC | 20.86953200 | -156.71791100 | 64.95 | 3433.96 |
| 32317e | 2017 | late | 2 | MC | 20.82909200 | -156.74476600 | 74.04 | 8328.45 |
| 13018A | 2018 | early | 3 | MCE | 20.85273700 | -156.75326500 | 73.30 | 7553.26 |
| 13018c | 2018 | early | 1 | S | 20.84884600 | -156.74501000 | 69.37 | 7002.09 |
| 13018d | 2018 | early | 1 | S | 20.84244700 | -156.72891200 | 58.11 | 6126.05 |
| 13018E | 2018 | early | 1 | S | 20.82567200 | -156.66851800 | 56.79 | 2372.29 |
| 2218a | 2018 | early | 3 | MUL | 20.83559400 | -156.74881000 | 51.77 | 8173.48 |
| 2318a | 2018 | early | 3 | MUL | 20.86517900 | -156.73878500 | 64.99 | 5633.97 |
| 2318b | 2018 | early | 4 | MUL | 20.85552000 | -156.74008200 | 70.12 | 6189.07 |
| 2318c | 2018 | early | 4 | MUL | 20.85552000 | -156.74008200 | 70.12 | 6189.07 |
| 2418a | 2018 | early | 1 | S | 20.84991600 | -156.75509600 | 73.24 | 7861.70 |
| 2618a | 2018 | early | 2 | D | 20.87583400 | -156.76025400 | 46.22 | 7584.24 |
| 2618b | 2018 | early | 2 | D | 20.88297300 | -156.74942000 | 39.37 | 6427.90 |
| 2618c | 2018 | early | 4 | MCEE | 20.87110500 | -156.74285900 | 70.36 | 5867.73 |
| 22118a | 2018 | mid | 2 | D | 20.85725400 | -156.69171100 | 42.64 | 2010.22 |
| 22118b | 2018 | mid | 3 | MUL | 20.85312100 | -156.69895900 | 43.30 | 2890.71 |
| 22218a | 2018 | mid | 3 | MCE | 20.85971800 | -156.71879600 | 71.68 | 4067.06 |
| 22218B | 2018 | mid | 1 | S | 20.84944300 | -156.75906400 | 69.72 | 8255.10 |
| 22418A | 2018 | mid | 2 | D | 20.83451100 | -156.70752000 | 65.62 | 4790.44 |
| 22418B | 2018 | mid | 3 | MCE | 20.83569700 | -156.69455000 | 61.55 | 3659.96 |
| 22518A | 2018 | mid | 2 | D | 20.81597900 | -156.68589800 | 62.48 | 4475.42 |
| 22518B | 2018 | mid | 1 | S | 20.81685100 | -156.67799400 | 65.56 | 3724.83 |
| 22518C | 2018 | mid | 3 | MUL | 20.80583200 | -156.63890100 | 52.00 | 1444.72 |
| 31918a | 2018 | late | 3 | MCE | 20.80616835 | -156.70045270 | 72.45 | 6331.88 |
| 12718a_o | 2018 | early | 2 | D | 20.88163000 | -156.77110300 | 49.41 | 8683.08 |
| 32018a | 2018 | late | 3 | MCE | 20.83607800 | -156.76001500 | 73.33 | 9080.82 |
| 12718c_o | 2018 | early | 2 | D | 20.87155900 | -156.76521300 | 53.61 | 8151.46 |
| 32218c | 2018 | late | 1 | S | 20.85430500 | -156.71809400 | 73.08 | 4402.05 |
| 12718d_o | 2018 | early | 1 | S | 20.86585600 | -156.76785300 | 70.98 | 8532.58 |
| 12718e_o | 2018 | early | 2 | D | 20.81168200 | -156.74012800 | 55.60 | 9020.46 |
| 12718f_o | 2018 | early | 3 | MUL | 20.81495100 | -156.73568700 | 66.77 | 8434.00 |
| 12718g_o | 2018 | early | 2 | D | 20.80817400 | -156.72348000 | 66.14 | 7943.95 |
| 12718h_o | 2018 | early | 3 | MUL | 20.80852900 | -156.72645600 | 56.49 | 8139.00 |
| 12718i_o | 2018 | early | 1 | S | 20.80868000 | -156.71827700 | 75.22 | 7529.13 |
| 12718j_o | 2018 | early | 3 | MCE | 20.80296700 | -156.70533800 | 65.95 | 6950.64 |
| 12718b_o | 2018 | early | 3 | MUL | 20.87363400 | -156.76492300 | 40.09 | 8092.29 |
| 21118a_o | 2018 | mid | 4 | MUL | 20.87413600 | -156.76736500 | 51.58 | 8338.87 |
| 21118b_o | 2018 | mid | 2 | D | 20.87879900 | -156.77052300 | 45.70 | 8629.65 |
| 21118c_o | 2018 | mid | 5 | MUL | 20.87640200 | -156.76698300 | 51.07 | 8277.00 |
| 21118d_o | 2018 | mid | 1 | S | 20.86492900 | -156.76696800 | 73.07 | 8464.77 |
| 21118e_o | 2018 | mid | 1 | S | 20.81195600 | -156.73318500 | 53.59 | 8423.16 |
| 21118f_o | 2018 | mid | 1 | S | 20.81081600 | -156.73374900 | 56.68 | 8547.48 |
| 21118g_o | 2018 | mid | 3 | MCE | 20.81024700 | -156.72860700 | 54.57 | 8176.94 |
| 21118i_o | 2018 | mid | 1 | S | 20.80301700 | -156.70858800 | 72.83 | 7238.36 |
| 32018a_2_o | 2018 | late | 3 | MCE | 20.83607800 | -156.76001500 | 73.33 | 9080.82 |

Abbreviations used for group composition: MC – maternal female + calf, MCE - maternal female, calf + escort, MCEE - maternal female, calf, > 1 escort, S – single, D – Dyad, M- Multiple adult group.

Where “o” is included in pod ID, group was encountered during mid channel surveys.

Table S3: Summary of encounter rates during transect-based surveys of the Au’Au Channel, 2008 – 2018.

| year | season | distance | N of whales | Total N of groups | N of calf groups | N of adult groups |
| --- | --- | --- | --- | --- | --- | --- |
| 2008 | Late | 270.50 | 113 | 44 | 27 | 17 |
| 2009 | Late | 355.30 | 179 | 77 | 42 | 35 |
| 2010 | Late | 105.30 | 64 | 27 | 17 | 10 |
| 2013 | Early | 60.60 | 115 | 45 | 28 | 17 |
| 2013 | Mid | 54.90 | 105 | 47 | 23 | 24 |
| 2013 | Late | 52.50 | 59 | 23 | 17 | 6 |
| 2014 | Early | 26.10 | 16 | 8 | 3 | 5 |
| 2014 | Mid | 31.90 | 41 | 14 | 9 | 5 |
| 2014 | Late | 34.20 | 26 | 12 | 9 | 3 |
| 2016 | Late | 50.30 | 8 | 4 | 3 | 1 |
| 2017 | Early | 53.86 | 29 | 14 | 1 | 13 |
| 2017 | Mid | 47.90 | 49 | 20 | 10 | 10 |
| 2017 | Late | 44.90 | 15 | 7 | 6 | 1 |
| 2018 | Early | 48.50 | 29 | 12 | 2 | 10 |
| 2018 | Mid | 47.70 | 20 | 9 | 2 | 7 |
| 2018 | Late | 49.69 | 7 | 3 | 2 | 1 |

Table S4: Encounter rates for line surveys conducted in the mid-channel waters of the Au’Au Channel, Maui, 2018

| Season | Date | Distance travelled (km) | Number of individuals (groups) sighted | Number of mother-calf groups sighted | Encounter rates all whales (groups) (km ^-1^) | Encounter rates (calf groups km^-1^) |
| --- | --- | --- | --- | --- | --- | --- |
| Early | 1/27/18 | 12.8 | 23 (10) | 1 | 1.79 (0.78) | 0.08 |
| Mid | 2/11/18 | 12.8 | 18 (8) | 1 | 1.40 (0.62) | 0.08 |
| Late | 3/17/18 | 12.8 | 3 (1) | 1 | 0.23 (0.08) | 0.08 |
| Totals |  | 38.4 | 43 (19) | 3 | 1.12 (0.49) | 0.08 |
